# Towards the Irving-Kirkwood limit of the mechanical stress tensor

**DOI:** 10.1063/1.4984834

**Published:** 2017-06-14

**Authors:** E. R. Smith, D. M. Heyes, D. Dini

**Affiliations:** 1 Department of Civil Engineering, Imperial College London, Exhibition Road, South Kensington, London SW7 2AZ, United Kingdom; 2 Department of Mechanical Engineering, Imperial College London, Exhibition Road, South Kensington, London SW7 2AZ, United Kingdom

## Abstract

The probability density functions (PDFs) of the local measure of pressure as a function of the sampling volume are computed for a model Lennard-Jones (LJ) fluid using the Method of Planes (MOP) and Volume Averaging (VA) techniques. This builds on the study of Heyes, Dini, and Smith [J. Chem. Phys. **145**, 104504 (2016)] which only considered the VA method for larger subvolumes. The focus here is typically on much smaller subvolumes than considered previously, which tend to the Irving-Kirkwood limit where the pressure tensor is defined at a point. The PDFs from the MOP and VA routes are compared for cubic subvolumes, $V=\ell^{3}$. Using very high grid-resolution and box-counting analysis, we also show that any measurement of pressure in a molecular system will fail to exactly capture the molecular configuration. This suggests that it is impossible to obtain the pressure in the Irving-Kirkwood limit using the commonly employed grid based averaging techniques. More importantly, below ℓ≈3 in LJ reduced units, the PDFs depart from Gaussian statistics, and for ℓ=1.0, a double peaked PDF is observed in the MOP but not VA pressure distributions. This departure from a Gaussian shape means that the average pressure is not the most representative or common value to arise. In addition to contributing to our understanding of local pressure formulas, this work shows a clear lower limit on the validity of simply taking the average value when coarse graining pressure from molecular (and colloidal) systems.

## I. INTRODUCTION

The stress, or a pressure tensor (PT), is a central property in continuum mechanics, defining the load in a structure or the evolution of a fluid. With the increasing interest in micro-fluidic devices and nano engineering, there is a need to develop computational tools for small scale systems. This requires the motions of individual molecules to be averaged so that they can be understood in terms of flow fields which can be measured by experiments and compared to continuum fluid theory. The purpose of the average quantities is both to understand the flow behavior in terms of macroscopic fields, such as velocity and stress, and to link these to continuum grid based methods. However, the pressure tensor (PT) remains the subject of a great deal of confusion and debate in the molecular dynamics literature.^1–9^ A detailed understanding of the time and spatial dependence of the PT fluctuations is essential in the context of nanofluidic research and molecular-to-continuum coupling simulations.^10^

The virial formula is well-established as the default way to get pressure in most bulk system simulations.^11^ However, a local pressure tensor is often needed, especially when the modelled system is spatially inhomogeneous, for example, where a liquid is next to a wall at equilibrium or driven out of equilibrium by an additional constraint such as the imposed shear flow. Irving and Kirkwood^12^ derived an exact expression for the pressure at a point in space using the Dirac delta functional, which is the starting point of a number of popular PT formulations proposed in the literature. The non-uniqueness of the local PT is attributable to at least three factors: (a) the choice of the spatially uniform reference pressure (“gauge”), (b) the interaction path between the molecules, and (c) the sampling volume.^4^ The first two factors can be removed in practice if the gauge pressure is arbitrarily set to zero and a linear path between molecules is assumed for the contour between the two molecules, which is consistent with the impressed force assumed by the use of Newton’s laws.^13,14^ This leaves the sampling volume as the primary variable, which is the focus of this work. To explore this, we evaluate the spatial integration of the Irving and Kirkwood^12^ Dirac delta functional over a volume. The advantage of adopting a formal spatial integration is that the result takes the same form as the equation of fluid dynamics written in the control volume (CV) form.^15^ Both grid-based and mesh-free method measurements could be used to course grain the molecular system; the two approaches essentially measure the same information in a different manner. However, grid based methods are preferred by the authors as they can be shown to match the continuum control volume equations exactly.^15^ Adopting the CV description, the validity of the conservation equations at a point is relaxed and replaced by the requirement that the conservation laws hold only in an average sense over the volume. The pressure can then be expressed in terms of the forces and fluxes which cross a boundary plane, essentially the Method of Planes (MOP) pressure,^16^ localized to a region of space.^17^ The volume average (VA) definition of the pressure tensor is obtained by assuming a constant value in a given control volume, effectively resulting in a weighted average of the pair interaction terms that are within the CV.^5,6^ While it can be shown that the MOP pressure definition exactly satisfies the equations of continuum fluid motion in the weakened form,^15^ it exhibits larger statistical fluctuations than the VA as is shown below. This is because the MOP only counts the discrete interactions crossing the volume boundary while the VA counts the fraction of the interaction line between the molecules inside the volume (i.e., a localized version of the virial method used in bulk pressure studies).

Both methods for the local pressure give, after sufficient time averaging, the same value irrespective of the volume size and shape (we investigated the relationship between the VA and MOP average pressure behavior in Refs. 18 and 19). In contrast, the pressure fluctuation characteristics of the various PT formulations can be quite different. To obtain clarity, it is convenient to consider the probability density function (PDF). The behavior of the PDF and second and higher moments of model Lennard-Jones fluids were explored by us in Heyes *et al.*^19^ The focus of that study was mainly on the extent to which the Gaussian statistics exhibited for large subvolumes containing many molecules extended to molecularly small and different shaped subvolumes. It was found that the range of applicability of the Gaussian form could be extended by the introduction of “effective” second order thermodynamic used to define the variance. In this current work, we go to smaller volumes where a marked departure is observed from the Gaussian behavior noted previously. A mesh of small subvolumes may be required to capture physical properties on very small scales, such as when a fluid is next to a wall. In fact, grid refinement is also an essential procedure in computational fluid dynamics^20^ to ensure that important fluid features are resolved correctly in the simulation without excessive and unnecessary resolution. In molecular simulations, grid refinement means we can explore the dynamics at the scale where effects of the molecular structure are dominant. The dynamics of particles at the level of molecular pores is very interesting, with eddying like motions being of particular interest, given the scales of the turbulent motion are typically assumed to be orders of magnitude larger.^21^ The resulting stresses due to in pore rotation and frustrated movements by molecular cages are explored here by defining a grid at the scale of the molecular pores. By going to smaller scales, we explore the impact of these motions. Similar considerations give an optimum level of coarse graining of molecular systems to obtain an effective continuous flow field from the individual molecular trajectories.

The fluctuation behavior is important to characterize and understand in the context of “molecular-to-continuum” coupling simulation methodology which requires a Molecular Dynamics (MD) region to provide pressure and other fields^22,23^ and to exchange with a continuum or hydrodynamically described region. Sufficient time and spatial averaging are required to minimize the effects of fluctuations of the passed information to the continuum region.^10,24^ Alternatively, these fluctuations can be preserved if they are also introduced in the continuum description of the system (the fluctuating hydrodynamics method assumes that they are Gaussian^25,26^). An understanding of the CV size dependence of the pressure PDFs could therefore ultimately be useful in devising more rigorous and computationally efficient coupling schemes. The focus of this study is to address these issues in the limit of zero volume, which has received little attention in the literature. This is perhaps surprising as the Irving and Kirkwood^12^ pressure tensor definition is only valid in the limit of zero volume. Understanding the volume size dependence of the statistical fluctuations of the system properties is central to the process of linking MD to a continuum description of the liquid. As will be explained, the effects of the microstructure of the liquid have a significant effect on the PDFs in this limit.

Details of the molecular dynamics simulation procedure and the definitions of the local pressure tensor are discussed in Sec. II. The theoretical outline of the measured quantities is discussed in Sec. III. In Sec. IV, the PDF behavior for a varying control volume size is presented in terms of a range of PDFs. The box counting fractal dimension is also considered in the later part of this section. A discussion of the results is given in Sec. V, followed by conclusions in Sec. VI.

## II. COMPUTATIONAL METHODOLOGY

In this work, molecular dynamics (MD) simulation was carried out using a Lennard-Jones pair potential

$$\Phi \left(r_{ij}\right)=4𝜖\left[{\left(\frac{\sigma }{r_{ij}}\right)}^{12}-{\left(\frac{\sigma }{r_{ij}}\right)}^{6}\right],\;r_{ij}\leq |r_{c}|,$$ (1)

where $r_{ij}\;\equiv \;r_{i}-r_{j}$ is the difference between the vectorial position of atom *i* located at $r_{i}$ and atom *j* located at $r_{j}$. The molecular diameter is σ and 𝜖 is the characteristic energy of the interaction. All molecules had the same mass, *m*, so the terms “velocity” and “momentum” can be used interchangeably. The truncation distance in the MD simulations was *r*_*c*_ = 2.5. The system consisted of 16 384 molecules surrounded by periodic boundaries in all three Cartesian directions. The reduced density was ρ = 0.8, and the sidelength of the simulation cell or “domain” was *L* = 27.36. All quantities are given in terms of the basic units, σ, 𝜖, and *m*. The equations of motion were integrated using the Verlet leapfrog integrator.^27^ An initialization stage of 100 000 time steps of magnitude, Δt = 0.005, was performed in the NVT ensemble using the Nosé-Hoover thermostat^28,29^ at a temperature *T* = 1.0. The simulation was then restarted from the final configuration and all statistics presented in this work were collected in an NVE ensemble production simulation.

The whole domain was divided into a space filling lattice or mesh of *N*_*cells*_ cells for which the various properties were calculated. The procedure adopted was to increase progressively the number of cells in the domain (i.e., resolution) to capture more of the fine detail. No time averaging was employed before entering data in the histograms used to construct PDFs in this work. Instantaneous samples at successive time steps are taken until the PDF distribution histogram converges to a time independent limiting form. With diminishing cell volume, there are more cells and so more samples are accumulated at each time step, resulting in convergence to a limiting PDF in fewer time steps.

The range of cell sizes and total number used in the simulations and subsequent analysis are summarized in Table I.

**TABLE I.** The range of grid cell sizes used in this work. In each case, the same MD simulation was repeated and the simulation cell volume was split into varying numbers of cells of sidelength (ℓ). The dots in the table in the 7-th column denote the existence of intervening values between 18 and 279, in multiples of 9 cells per side.

Cells per side	1	2	4	9	18	⋯	279	288
Cell side length (ℓ)	27.36	13.68	6.84	3.04	1.52	⋯	0.098	0.095
Total number of cells	1	8	64	729	5832	⋯	21 717 639	23 887 872

## III. PRESSURE TENSOR THEORY

For completeness, the equations defining the various local PT measures are given in this section. The velocity average in a control volume is given by the integral of the Irving and Kirkwood^12^ expression

$$\underline{u}=\frac{1}{M_{I}}\int_{V}\overset{N}{\underset{i=1}{\sum }}m_{i}{\underline{v}}_{i}\delta \left(\underline{r}-{\underline{r}}_{i}\right)dV=\frac{1}{M_{I}}\overset{N}{\underset{i=1}{\sum }}m_{i}{\underline{v}}_{i}{𝜗}_{i},$$ (2)

where $M_{I}$ is the sum of mass of all molecules in the cell, and $M_{I}=\sum_{i=1}^{N}m_{i}{𝜗}_{i}$. The variable ${𝜗}_{i}$ is a functional with a value one when a molecule *i* is in the control volume and zero otherwise, defined formally in Smith *et al.*^15^ The instantaneous center of mass of a given control volume is calculated using

$$\underline{R}=\frac{1}{M_{I}}\overset{N}{\underset{i=1}{\sum }}m_{i}\left({\underline{r}}_{i}-\underline{r}\right){𝜗}_{i}.$$ (3)

The center of mass is a useful parameter which can be related to the pressure PDF, as discussed below. The Irving and Kirkwood^12^ equation for the pressure tensor is

$$\begin{array}{rcl} \overset{IK}{\underset{\_}{\underline{\Pi }}} & = & \overset{N}{\underset{i=1}{\sum }}\frac{1}{m_{i}}{\underline{p}}_{i}{\underline{p}}_{i}\delta \left(\underline{r}-{\underline{r}}_{i}\right)\\ & & +\;\frac{1}{2}\overset{N}{\underset{i=1}{\sum }}\overset{N}{\underset{j\neq i}{\sum }}{\underline{r}}_{ij}{\underline{f}}_{ij}\int_{0}^{1}\delta \left(\underline{r}-{\underline{r}}_{i}+\lambda {\underline{r}}_{ij}\right)d\lambda , \end{array}$$ (4)

where the momenta ${\underline{p}}_{i}=m_{i}{\underline{v}}_{i}-\underline{u}$, *m* is the mass of the molecule, ${\underline{r}}_{ij}$ is the pair separation vector, and ${\underline{f}}_{ij}$ is the pair force. The spatial integral of these equations over a volume in space gives the control volume (CV) form of the pressure tensor

$$\int_{V}\underline{\underline{\Pi }}dV=\overset{N}{\underset{i=1}{\sum }}\frac{1}{m_{i}}{\underline{p}}_{i}{\underline{p}}_{i}{𝜗}_{i}+\frac{1}{2}\overset{N}{\underset{i=1}{\sum }}\overset{N}{\underset{j\neq i}{\sum }}{\underline{r}}_{ij}{\underline{f}}_{ij}\int_{0}^{1}{𝜗}_{\lambda }d\lambda ,$$ (5)

where ${𝜗}_{\lambda }$ is a functional with a value one when a part of the interaction between *i* and *j* is in the control volume and zero otherwise. The fraction of the line in the cell is then $\int_{0}^{1}{𝜗}_{\lambda }d\lambda$. In order to get the average PT in a volume, we assume $\int_{V}\underline{\underline{\Pi }}dV\approx \overset{VA}{\underset{\_}{\underline{\Pi }}}V$ and then Eq. (5) defines the so-called volume average pressure. The virial expression^30^ for the pressure tensor, $\overset{\text{VIRIAL}}{\underset{\_}{\underline{\Pi }}}$, is simply Eq. (5) when the averaging volume is the whole simulation domain

$$\overset{VIRIAL}{\underset{\_}{\underline{\Pi }}}V=\overset{N}{\underset{i=1}{\sum }}\frac{1}{m_{i}}{\underline{p}}_{i}{\underline{p}}_{i}+\frac{1}{2}\overset{N}{\underset{i=1}{\sum }}\overset{N}{\underset{j\neq i}{\sum }}{\underline{r}}_{ij}{\underline{f}}_{ij}.$$ (6)

Expressing Eq. (5) in terms of surface fluxes gives the pressure over all six faces of the cube^15^

$$\oint_{S}\underline{\underline{\Pi }}\cdot \;d\underline{S}=\overset{N}{\underset{i=1}{\sum }}\frac{1}{m_{i}}{\underline{p}}_{i}{\underline{p}}_{i}\cdot d{\underline{S}}_{i}+\frac{1}{2}\overset{N}{\underset{i=1}{\sum }}\overset{N}{\underset{j\neq i}{\sum }}{\underline{f}}_{ij}\underline{n}\cdot d{\underline{S}}_{ij},$$ (7)

where $\underline{n}=\left[1,1,1\right]$ to allow a sum over all faces. The Method of Planes (MOP)^16^ form of stress localized to a surface^17^ is obtained by assuming an average value on any one of the faces of the cube, for example, taking the top here as (denoted by a “+” superscript) ∮Sx+Π__⋅ dS_x+≈Π_MOPAxx whose normal is in the *x* direction

Π_MOPAxx=∑i=1N1mip_ipxidSxi++12∑i=1N∑j≠iNf_ijdSxij+, (8)

where $dS_{xi}^{+}$ captures the molecule, *i*, as it crosses the surface and $dS_{xij}^{+}$ is 1 when the intermolecular interaction between *i* and *j* crosses the surface and 0 otherwise.^15^ The first term on the right hand side of Eqs. (4)–(8) is the kinetic part of the pressure tensor, and the second is the “interaction,” or “configurational” contribution to the pressure tensor for a given control volume *I*. The focus of this work is the configurational pressure, a quantity only obtainable from the molecular simulation by simulating the configuration of molecules evolving through time. For the VA definition, the interaction component is

$$\overset{VA}{\underset{\_}{\underset{\_}{\Pi^{C}}}}=\frac{1}{2V}\overset{N}{\underset{i=1}{\sum }}\overset{N}{\underset{j\neq i}{\sum }}{\underline{r}}_{ij}{\underline{f}}_{ij}\int_{0}^{1}{𝜗}_{\lambda }^{I}d\lambda .$$ (9)

and for the MOP considering the top *x* surface,

$$\overset{MOP}{\underset{\_}{\Pi_{x}^{C}}}=\frac{1}{2A_{x}}\overset{N}{\underset{i=1}{\sum }}\overset{N}{\underset{j\neq i}{\sum }}{\underline{f}}_{ij}dS_{xij}^{+}.$$ (10)

**FIG. 1.**
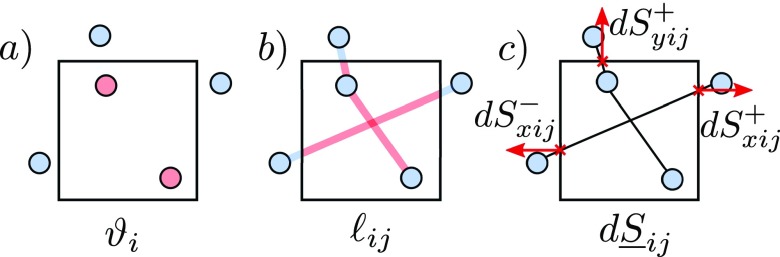
Physical meaning of some of the variables used in defining the PT. Key: (a) Points inside a volume, (b) illustrates the fraction of the line in the volume, which is relevant for VA, and (c) shows pair interactions crossing the various volume surfaces and in particular, the normal components of these vectors (shown as red arrows), relevant for the direct pressure components of the MOP.

The relationship between these two forms of the pressure tensor is show schematically in Figs. 1(b) and 1(c). The definitions in Eqs. (9) and (10) represent quite different mathematical procedures, emphasizing distinct aspects of the pair interactions and their capture in the subvolume. Equation (9) sums the terms, ${\underline{r}}_{ij}{\underline{f}}_{ij}$, weighted by the fraction of the length of the line between the two molecules inside the cell, $\int_{0}^{1}{𝜗}_{\lambda }^{I}d\lambda$, and assigns them to control volume *I*. Equation (10) takes the pair force, ${\underline{f}}_{ij}$, for all intermolecular interactions and assigns it to cell *I* if the top *x* is crossed by that interaction.

The velocity and center of mass depend only on the molecular position as show by Fig. 1(a). The local pressure tensor definition requires some aspects of the interaction vector between all the molecular pairs to be considered. As the calculation of the PT involves averaging over a volume or surface, the spatial resolution of the grid has a strong effect on the stress profile for small grid cell volumes. The continuum PT is, strictly speaking, only defined in the limit of zero volume.^31^ In this limit, the VA and MOP pressure definitions return to the Irving and Kirkwood^12^ form. That is, for the VA Eq. (9),

$$\underset{V\to 0}{\lim}\overset{VA}{\underset{\_}{\underset{\_}{\Pi^{C}}}}=\frac{1}{2}\overset{N}{\underset{i=1}{\sum }}\overset{N}{\underset{j\neq i}{\sum }}{\underline{r}}_{ij}{\underline{f}}_{ij}\int_{0}^{1}\underset{V\to 0}{\lim}\left[\frac{{𝜗}_{\lambda }^{I}}{V}\right]d\lambda =\overset{IK}{\underset{\_}{\underset{\_}{\Pi_{C}}}},$$ (11)

and for the MOP on the *x* surface from Eq. (10),

$$\underset{A_{x}\to 0}{\lim}\overset{MOP}{\underset{\_}{\Pi_{x}^{C}}}=\frac{1}{2}\overset{N}{\underset{i=1}{\sum }}\overset{N}{\underset{j\neq i}{\sum }}{\underline{f}}_{ij}\underset{A_{x}\to 0}{\lim}\frac{dS_{xij}^{+}}{A_{x}}=\overset{IK}{\underset{\_}{\underset{\_}{\Pi_{C}}}},$$ (12)

using the definition of the Dirac delta functional

$$\delta \left(x\right)\equiv \underset{\Delta x\to 0}{\lim}\frac{\left[H\left(x+\Delta x\right)-H\left(x-\Delta x\right)\right]}{\Delta x}$$ (13)

as both $dS_{xij}^{+}∕A_{x}$ and ${𝜗}_{\lambda }^{I}∕V$ are in the form of Eq. (13), i.e., the difference between two Heaviside functionals divided by their separation. In this work, we use probability density functions (PDFs) of subvolume pressure to investigate the approach to the limit of infinitesimally small cell volume. No time averaging was employed in defining the configurational pressure values from Eqs. (9) and (10) for the PDF, only instantaneous cell spatial averaging. The first moment of the PDFs is the mean value which would have been obtained by time averaging Eqs. (9) and (10), so the presented PDFs provide insight into all results which contribute to the definition of time averaged pressure. The collection of instantaneous samples is also meaningful in itself because the conservation laws for mass, momentum, and energy are exact even without any temporal or ensemble averaging.^15,32^

## IV. RESULTS

**FIG. 2.**
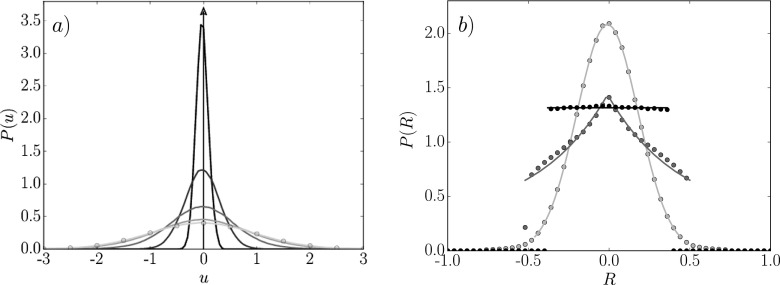
(a) PDF of velocity for cell sizes ℓ=4.69, ℓ=2.33, ℓ=1.55, ℓ=1.16, ℓ=0.932, shown in gray, darkest to lightest, the velocity PDF of the individual molecules (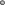), and an arrow showing the thermodynamic limit. (b) PDF of the center of mass of the molecules in the control volume with MD results shown by points and Gaussian, Laplace/uniform combination and uniform fits shown by lines ℓ = 1.52 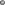 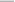
(light gray), ℓ = 1.0 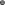 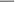
(gray), and ℓ = 0.76 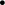 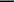
(black), respectively.

Both the center of mass and velocity PDFs are presented for a range of cell volumes in Fig. 2. Figure 2(a) shows the cell velocity PDF for several cell sizes, which is Gaussian in all presented cases. The larger the cell the smaller the cell velocity fluctuations and the narrower the distribution; the theoretical thermodynamic limit is shown by an arrow in Fig. 2(a). As cell sizes become smaller, the distribution gets wider and eventually has only a single molecule per bin with a velocity distribution which matches that of the molecules themselves [shown by circles on Fig. 2(a)]. The PDF of the center of mass cell measurements from Eq. (3) can be Gaussian, a combination of uniform and Laplacian, and uniform in the form as shown in Fig. 2(b). The distribution of center of mass for large cell volumes is Gaussian, which is a manifestation of the central limit theorem with large numbers of molecules. A PDF for a cell volume of ℓ=1.52 is well fitted by the Gaussian distribution

$$P_{G}\left(x,\mu ,s\right)=\frac{1}{s\sqrt{2\pi }}e^{-\frac{{\left(x-\mu \right)}^{2}}{2s^{2}}}$$ (14)

as shown in Fig. 2(b). The coefficients μ and *s* are the mean and standard deviation of *x*, respectively. With decreasing cell size, fewer molecules can occupy a cell (based on an average density) because of excluded volume interactions. If the cell is small enough to contain only one molecule, the PDF is flat (indicating a uniform distribution) as there is no preferential location for that molecule with respect to the center of the cell. A uniform distribution is fitted for ℓ=0.76 in Fig. 2(b). For ℓ=1.0, cases arise in which some cells contain a single molecule and some contain two molecules. Two molecules in a cell can only fit when they are at opposite ends of the cell, due to excluded volume effects, and the center of mass will be close to zero (i.e., located at the center of the cell). With two molecules in a cell, zero is the most likely value and any non-zero center of mass requires the molecules to be forced together beyond the equilibrium separation. This gives rise to a Laplace (also called a double exponential) shape to the PDF

$$P_{Lap}\left(x,\mu ,b\right)=\frac{1}{2\;b}\exp\left(-\frac{\left|x-\mu \right|}{b}\right).$$ (15)

This distribution occurs because, with two molecules in a cell, any departure from a non-zero center of mass is exponentially less likely. The intermediate case as shown in Fig. 2(b) is seen to be fitted well by the superposition of a uniform distribution and a Laplace distribution representing the combination of single and pairs of molecules in the volumes.

**FIG. 3.**
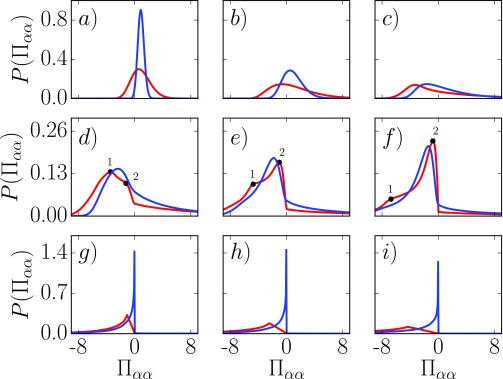
PDFs for VA (blue) and MOP (red) where the top row shows Gaussian and skewed Gaussian PDFs labeled (a)–(c) for ℓ=6.84,3.04,1.52, respectively; the middle row is the two peak region for the MOP PDFs and skewed PDFs for VA (d)–(f) with sizes ℓ=1.01,0.76,0.61, respectively; and the bottom row is the limiting cases, showing (g)–(i) which are ℓ=0.19,0.127,0.095, respectively.

The PDFs of the pressure tensor are considered now. The shear (off diagonal) components of the pressure tensor are zero on average. As the current work focuses on an equilibrium system, the direct pressure PDFs are of more interest and the shear stress PDFs will not be considered. The pressure is the trace of the pressure tensor of Eq. (9) or the force components normal to the surface in Eq. (10). Figure 3 shows the VA and MOP PDFs for a range of grid cell volumes. For large cells with sidelengths of ℓ ≥ 13.7, the pressure PDF is seen to be well fitted by a Gaussian for both MOP and VA methods, as shown in the Appendix. For any grid cell volume larger than this value, the standard deviation tends towards zero.^19^ For smaller volumes than ℓ≈6.84, the Gaussian distributions start to become skewed to the left as shown in Figs. 3(a) and 3(b). This type of PDF can be fitted using a skewed Gaussian of the form

$$P_{Gskew}\left(x,\mu^{\prime },s^{\prime },\alpha \right)=2P_{G}\left(x,\mu^{\prime },s^{\prime }\right)\int_{-\infty }^{\alpha x}P_{G}\left(x^{\prime },\mu^{\prime },s^{\prime }\right)dx^{\prime },$$ (16)

where $\mu^{\prime },s^{\prime },\alpha$ are the parameters which can be obtained by fitting to the simulation data. For the skewed distribution shown in the frames, Figs. 3(a) and 3(b), the distribution for the cell size of ℓ=6.84 and 3.04 is fitted quite well by the analytic form in Eq. (16) for both VA and MOP pressures as shown in the Appendix. For ℓ=1.52, shown in Fig. 3(c), the skewed Gaussian is only a good fit to the VA case, again demonstrated explicitly in the Appendix.

Figures 3(d)–3(f) present the pressure PDFs for ℓ = 1.01,0.76, and 0.608. Over this range, the ratio of volume to surface area (V∕A=ℓ) goes below unity and, perhaps significantly, the cell sidelength becomes less than the minimum separation in the LJ potential, $2^{1∕6}\;\approx \;1.1225$. More extreme PDF shapes are evident with different behaviors for the VA and MOP which reflect the consequences of their different definitions in Eqs. (9) and (10), respectively. The molecular forces are divided by smaller volumes or areas which also contribute to the appearance of these extreme and anomalous PDF features.

Notably, the positive tail is extremely long, with large values of pressure observed far outside the range of the plot, with some greater than fifty. One might expect, however, that the repulsive interactions are relatively rare compared to the more numerous contributions from the attractive part of the potential, which produces a long negative pressure tail as well.

Also evident in Figs. 3(d)–3(f) is a double peak in the MOP pressure distribution. In Fig. 3(d), the peak labeled 1 coincides with the Gaussian peak evident in (a)–(c) but is more skewed. A second peak labeled 2 also starts to become apparent in the MOP distributions of Fig. 3(d). For the next Fig. 3(e), the Gaussian peak 1 has shifted further to the left and the second peak 2 is now larger. For the smallest volume, ℓ = 0.608 in Fig. 3(f), the Gaussian peak 1 is almost insignificant while the new peak 2 is now dominant. This same double peak is not observed for the volume average distribution of Figs. 3(d)–3(f); however, the distribution also appears to shift left followed by a move back to the right. This suggests that a similar change may occur for VA PDFs as the volume becomes smaller, with the two peaks obscured by the definition of the VA pressure, Eq. (9); the continuous variation of line fractions gives a continuous range of values instead of the binary surface crossing monitored using the MOP.

For the volumes shown in Figs. 3(g)–3(i), with volumes smaller than ℓ=0.2, fewer interactions are sampled per cell; thus, the peaks of the PDFs show a shift toward zero. This is especially the case for the VA pressure, where increasingly small parts of the interaction line between pairs of the molecules in each box define the distribution of stresses. The result is a PDF that is dominated entirely by near-zero values similar in shape to an extreme value distribution. Once in the limit, where most boxes have a single interaction, (e.g., ℓ=0.179 in Fig. 3), the location of the VA dominant peaks stays fairly constant. The MOP distribution peak also decreases and shifts right for ℓ=0.2 to ℓ=0.095 in Figs. 3(g)–3(i). Once the volume is small enough to sample only a single intermolecular interaction, the MOP distribution shifts to the left. This is because the one interaction is divided by an increasingly small area. The change in the peak location is linearly proportional to an area for ℓ<0.152, as shown in the Appendix. The peak in the MOP distribution also corresponds to a minor peak observable in the VA distribution, although not clear on the scale of Figs. 3(g)–3(i). This suggests that the MOP peaks measuring single molecular interactions are present in both distributions but less apparent in the VA case due to numerous small partial line contributions. It is clear that in the limit of small volumes, single interaction statistics and increasingly small parts of intermolecular contributions define the distributions of pressure.

Even for the very small cell volumes, the VA and MOP PDFs do not show evidence of convergence to a constant shape. In fact, for ℓ=0.095, the highest resolution considered, 23 × 10^6^ cells are required to fill the whole domain (c.f. *N* = 16 384 molecules) and the PDF, is still changing even at that resolution. Considering that the molecular system has only 6*N* = 98 304 degrees of freedom, grid averaging is clearly inefficient as a means of measuring the pressure of the system. It appears, therefore, to be impractical to refine the grid until all intermolecular interaction lines can be exactly represented by very small bins. As for a fractal object, it is not possible to describe the system exactly at *any* level of grid refinement. This suggests that the fractal dimension of the network of interparticle distance vectors may provide insights into the convergence characteristics of the pressure PDFs. One type of fractal dimension is that the box counting, *D*_0_, can be obtained from the ℓ→0 limit

$$D_{0}=\underset{\ell \to 0}{\lim}\frac{\ln\left(M\left(\ell \right)\right)}{\ln\left(1∕\ell \right)},$$ (17)

where *M* is the number of boxes which has a non-zero value for the VA or MOP pressure. The interactions act along lines between molecules which can be thought of as forming a three dimensional “haystack” structure. The number of boxes required to encompass a single line is inversely proportional to the box size, so one might expect M(ℓ)=a∕ℓ, where *a* is a geometry-related constant. Taking the logarithm of both sides of M(ℓ)=a∕ℓ and upon rearrangement

$$\frac{\ln\left(M\left(\ell \right)\right)}{\ln\left(1∕\ell \right)}=1+\frac{\ln\left(a\right)}{\ln\left(1∕\ell \right)}.$$ (18)

**FIG. 4.**
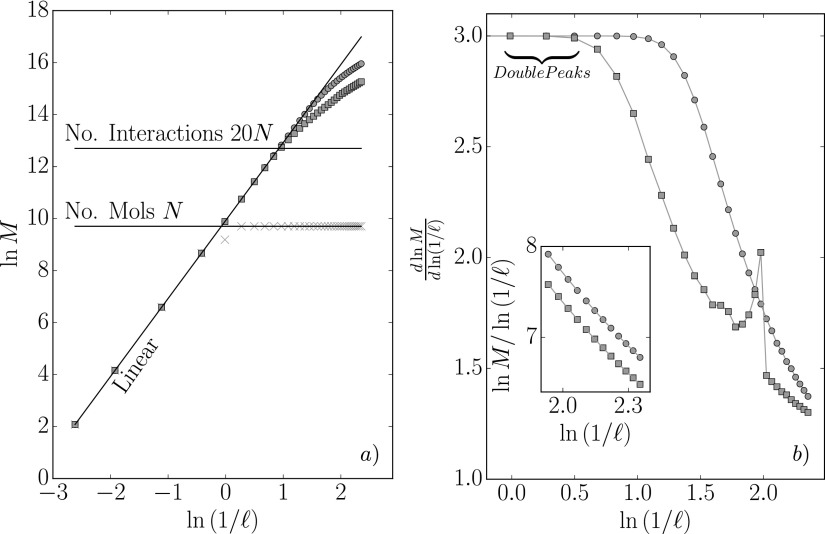
Box counting analysis of the number of cells, *M*, with at least one molecule inside the volume (×), some part of the pair interaction in the volume (related to VA, 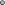), or some part of the interaction crossings the surface of the volume (related to MOP, 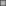). (a) shows the box sidelength vs. number of cells with horizontal lines at *N* and approximate number of interactions 20*N* as well as a log-linear line ln(M)=ln(1∕ℓ). (b) shows the slope of ln(M) with respect to ln(1∕ℓ) which would give the box counting dimension in the limit of zero cell volume. Lines are included to guide the eye. The inset shows the ratio of lnM to ln(1∕ℓ) for the smallest twelve cells with a fitted line shown for groups of four using the equation $1+\ln\left(a\right)∕{\left[\ln\left(1∕\ell \right)\right]}^{d}$, where *a* and *d* are fitting parameters.

Equation (18) shows that for a single line lnM∕ln[1∕ℓ]→1 in the ℓ→0 limit. By extension, a system with many lines may be expected to tend to this limit, provided the grid resolution could be made high enough to track each part of every line with fine resolution. The ratio ln(M(ℓ)) to ln(1∕ℓ) has not reached a constant value and a fit to a function of the form $1+\ln\left(a\right)∕{\left[\ln\left(1∕\ell \right)\right]}^{d}$, where *a* and *d* are fitting parameters, does not provide an adequate fit over the full range of values. However, as ℓ changes, different values of *a* and *d* provide a good fit locally, similar to a local scaling exponent which is a characteristic of a multi-fractal system. The fitting values for the three smallest groups of four points shown in the inset of Fig. 4(b) are MOP *d* = [0.899, 0.936, 0.944], *a* = [11.9, 12.2, 12.30], and VA *d* = [0.891, 0.914, 0.932], *a* = [12.4, 12.7, 12.8] which shows *d* tending to unity as expected from Eq. (18). Figure 4 shows how the gradient, dlnM∕dln[1∕ℓ], changes as a function of ln[1∕ℓ], which reveals the slow convergence to a limiting case. In the neighborhood of ℓ=0.22, a notable feature is evident in the derivative of MOP box counting in Fig. 4(b). Two peaks are seen which resemble the radial distribution function (RDF), *g*(*r*), and reflect the molecular nature of the liquid structure on this scale. The appearance of this peak follows from the definition of the RDF, dM∝ρg(r)dℓ, as we are plotting varying bin size dℓ against the resulting change in counted interactions *dM*. As ℓ gets significantly smaller than unity, M(ℓ) starts to sample those regions of space that have few molecules owing to excluded volume interactions between nearest neighbors. This small ℓ region corresponds to the high *k* wavevector limit in X-ray scattering which is also dominated by the individual particle shape (through the form factor).^27^

In this section, both larger volumes and the ℓ→0 limit have been explored. In the limiting case, the box counting fractal dimension appears to be converging very slowly and the PDFs become dominated by few, and eventually, single intermolecular interactions crossing the grid cell. Despite using very small volumes, it is apparent that it is not practical to reduce the volume size to obtain a limiting case for the Irving and Kirkwood^12^ stress.

## V. DISCUSSION

In Sec. IV, we have shown that pressure PDFs are Gaussian for volumes larger than ℓ=6.0. They become skewed at ℓ≈3.0 and eventually exhibit a more complex behavior as the volume of the bins becomes much smaller than the volume of a molecule. The distributions measured here could be used directly in fluctuating hydrodynamics, where noise is traditionally sampled from a Gaussian distribution as a model for sub-grid fluctuations in continuum equations, e.g., the fluctuating Navier-Stokes.^25,26,33–35^

**FIG. 5.**
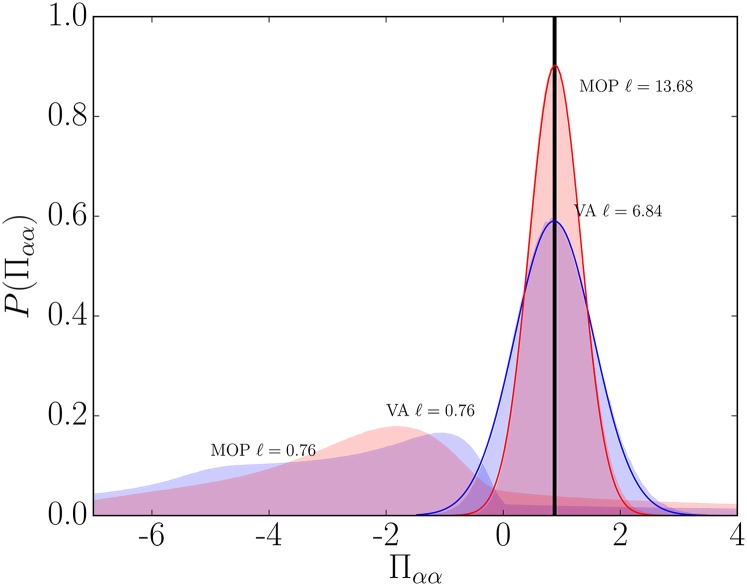
Distributions of pressure for the MOP (blue) and VA (red) for two bin sizes shown as filled areas, with Gaussian fits shown as solid lines. The thick black line shows the mean value of pressure in the system.

However, the departure from Gaussian statistics, highlighted in this work, has a far more profound implication. Quantities such as velocity and pressure are defined as the average over an ensemble of systems^12^ or multiple time steps if ergodicity is assumed.^27^ This average value is only meaningful if the ensemble of systems obeys Gaussian statistics.^36^ This point is emphasized in Fig. 5, where the mean pressure, Π=0.87, in the system is shown as a thick black line. For large volumes, it is clear that this mean value is a good representation of the normally distributed data. This is no longer true for small volumes; the PDFs for volumes of size ℓ=0.76 in Fig. 5 show that the average value is no longer a meaningful representation of measured pressure in the system. The highest peaks are less than zero and balanced by very long positive tails on the distribution. In fact, this is true even as the PDFs begin to skew below ℓ=3.04, see Figs. 3(b) and 3(c). Despite this, the first moment of these distributions still gives a similar mean, Π=0.87, for the MOP pressure and exactly the same value for the VA. This observation is important as the Irving and Kirkwood^12^ pressure assumes an ensemble average for the ℓ→0 limit in order to obtain equations consistent with continuum mechanics.

The continuum fluid equations describe the propagation of the averaged molecular quantities such as density, velocity, and pressure. In many cases where characteristic scales are large, the non-Gaussian nature of the molecular configurations is not important and the continuum equations describe the correct physics. The standard deviations of these Gaussian distributions give further detail in the form of temperatures (from velocity PDFs) and bulk modulus (from pressure).^19^ However, there are examples where continuum mechanics fails, including near the solid-liquid interfaces^37^ or at the three-phase contact line.^38^ Such local failure of the continuum equations is well know,^39^ with this behavior often localized to small distances from the wall.^37,40^ This may also have implications for turbulent-like flows, which have been simulated in molecular systems.^21^ When increasing the grid resolution, a multifractal behavior is observed and the pressure distribution departs from Gaussian, a property also observed in turbulent energy cascades.^41^ The results here suggest that even at the smallest scales, where dissipation is due to inter-molecular structure, there remains a scale dependence. In this case, as well as many others, it is apparent that further study of MD distributions can yield insight beyond simple averages.

## VI. CONCLUSIONS

Equilibrium molecular dynamics (MD) simulations have been carried out to explore the impact of the grid-averaging resolution on the pressure probability density functions (PDFs). Unlike the molecular velocities and positions, where a grid can be refined to exactly capture the information content of the underlying system, the configurational pressure is based on interactions which pass through the volume and is therefore inseparably linked to the resolution of the grid. Two measures of the local pressure are considered, Volume Averaging (VA) and the Method of Planes (MOP), as the averaging volume sizes are decreased towards the Irving and Kirkwood^12^ limit. For large volumes, e.g., ℓ≈6, the pressure PDFs for both VA and MOP are Gaussian. As the cell volume decreases in size, the PDF becomes skewed and with volume sidelengths below ℓ≈3.0, the pressure PDFs depart significantly from a Gaussian. This puts a very clear lower limit on the grid resolution where Gaussian statistics are valid and the mean and standard deviation of the pressure field are a meaningful concept. Cells of size ℓ≈3.0 are of the same length scale where the viscosity near a wall^37^ and in a liquid-vapor interface region^42^ manifests a departure from continuum models. The measured pressures using volumes at this scale and smaller are not trivially Gaussian, reflecting the underlying liquid structure and implying that the system cannot be treated as a continuum on that volume range. The Method of Planes (MOP) pressure PDFs for volume sizes around ℓ≈1 exhibit a bimodal distribution in what appears to be a competition between statistical averaging and microstructural effects. These two peaks are notably absent in the VA measure, where the averaging obscures more of the structural detail. For volumes much smaller than ℓ≈1, the PDFs are dominated by single interactions with extreme values. In these limits, only a single interaction will register in the cell, so the PDF shapes are dominated by small parts of a few interactions at most in the VA case and single forces divided by area in the MOP measure. Box counting is used to show that even with the smallest volume sizes studied, the limiting case is far from being reached. This suggests that any practical pressure calculation in a molecular or granular system will fail to fully capture the inter-molecular interactions. This work indicates that promising insights are possible by going beyond simple averaging, retaining essential molecular details through probability density functions. The use of mechanical measurements in local volumes results in a measure of pressure which is valid arbitrarily far from equilibrium, and the PDF techniques presented could shed light on the mechanism governing the dynamics of a shockwave or plastic deformation in materials.

## Figures and Tables

**FIG. 6.**
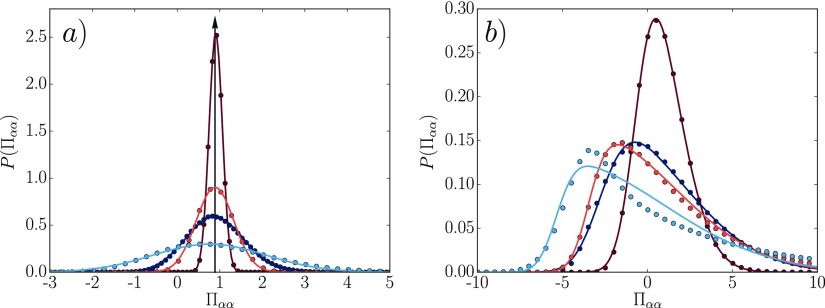
Molecular PDFs (a) with Gaussian fit for cells ℓ=13.7 VA (brown circle, brown line) and MOP cells (dark blue circle, dark blue line) and ℓ=6.8 VA (orange circle, orange line) and MOP (light blue circle, light blue line); and (b) with Skewed Gaussian fit for cells ℓ=3.04 VA (brown circle, brown line) and MOP cells (dark blue circle, dark blue line) and ℓ=1.52 VA (orange circle, orange line) and MOP (light blue circle, light blue line).

**FIG. 7.**
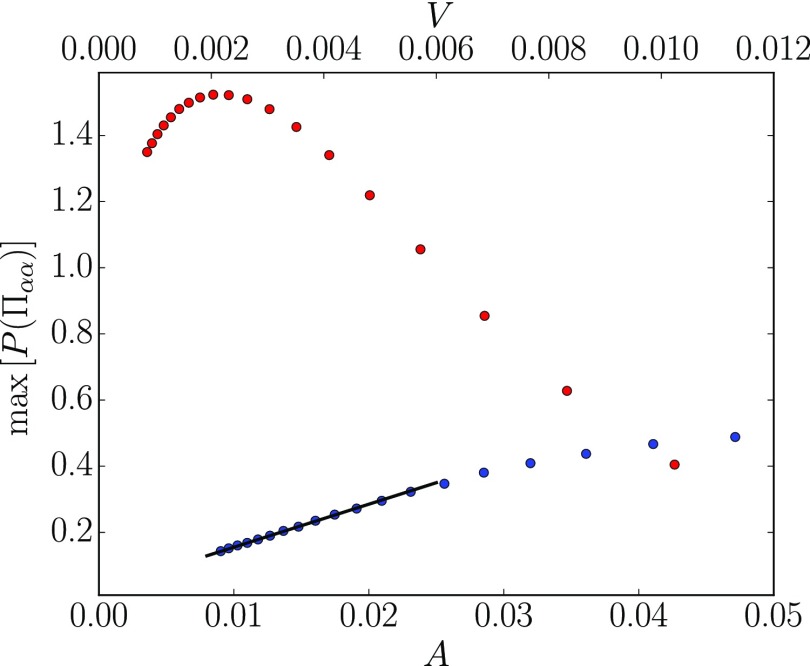
The location of the peak of the PDFs in the small volume limiting case for MOP (blue) and VA (red) plotted against area *A* and cell volume *V*, respectively. The black line is a line of the best fit to the points with form $max\left[P^{MOP}\left(\Pi_{\alpha \alpha }\right)\right]=13.0A+0.025$.

## APPENDIX: FITTING TO DISTRIBUTIONS

In this appendix, the Gaussian Eq. (14) and skewed Gaussian Eq. (16) are fitted to the histogrammed data using the Levenberg-Marquardt algorithm from Scipy version 0.15.0. The fits using the Gaussian distributions are shown in Fig. 6(a) and the skewed Gaussian distributions in Fig. 6(b). The Gaussian fits appear to be reasonable for ℓ=13.7 and ℓ=6.8, but as the distribution begins to skew at sidelengths of ℓ=3.04, a skewed Gaussian is seen to be a better fit to these distributions. The fits to the Gaussian distributions have the following mean and standard deviations, respectively: ℓ=13.7 VA 0.90, 0.16 MOP 0.87, 0.68. For ℓ=6.8, VA 0.89, 0.44 and MOP 0.76, 1.33. In all cases, the mean is fairly consistent except the MOP ℓ=6.8 value which is already starting to show a departure from the Gaussian behavior. The skewed Gaussian is seen to be a good fit for volumes of sidelength ℓ=3.04 and below, with the exception of the MOP PDF for sidelengths of ℓ=1.52. The moments of the skewed Gaussian, mean, standard deviation, and skewness, respectively, for ℓ=3.04 VA are 0.89, 1.44, 0.47 and MOP 0.80, 2.95, 0.75. For ℓ=1.52, VA are 0.55, 3.13, 0.89 and MOP are −0.43, 3.81, 0.92. Notice that the mean values are considerably different from the global mean below a cell size of three, as discussed in the main text.

A plot of the location of the peak of the distribution, Fig. 7, is shown here for the small volume limit. The maximum peak in the VA distribution of measured stress is plotted against volume (shown on the top axis). It is apparent that there is a clear trend in the peak location and that this has not converged to a limiting value. The MOP peak pressure value is plotted against area on the bottom axis and below A≈0.025 appears to be linearly proportional to the surface area, which is displayed by a line fit of the form $max\left[P^{MOP}\left(\Pi_{\alpha \alpha }\right)\right]=13.0A+0.025$.
